# Supplementary material for: DMRforPairs: identifying Differentially Methylated Regions between unique samples using array based methylation profiles
Source: BMC Bioinformatics. 2014 May 15;15:141. doi: 10.1186/1471-2105-15-141 (PMC4046028; doi:10.1186/1471-2105-15-141)
Supplement: Additional file 2 — DMRforPairs output for the comparison of A431-MCF7 and NA17018-NA17105. Please start from the HTML files in each folder. Available via the BMC Bioinformatics website. [file 1471-2105-15-141-S2.zip › 1394847754114233_MOESM2_ESM/A431_MCF7/figures/6690.pdf]

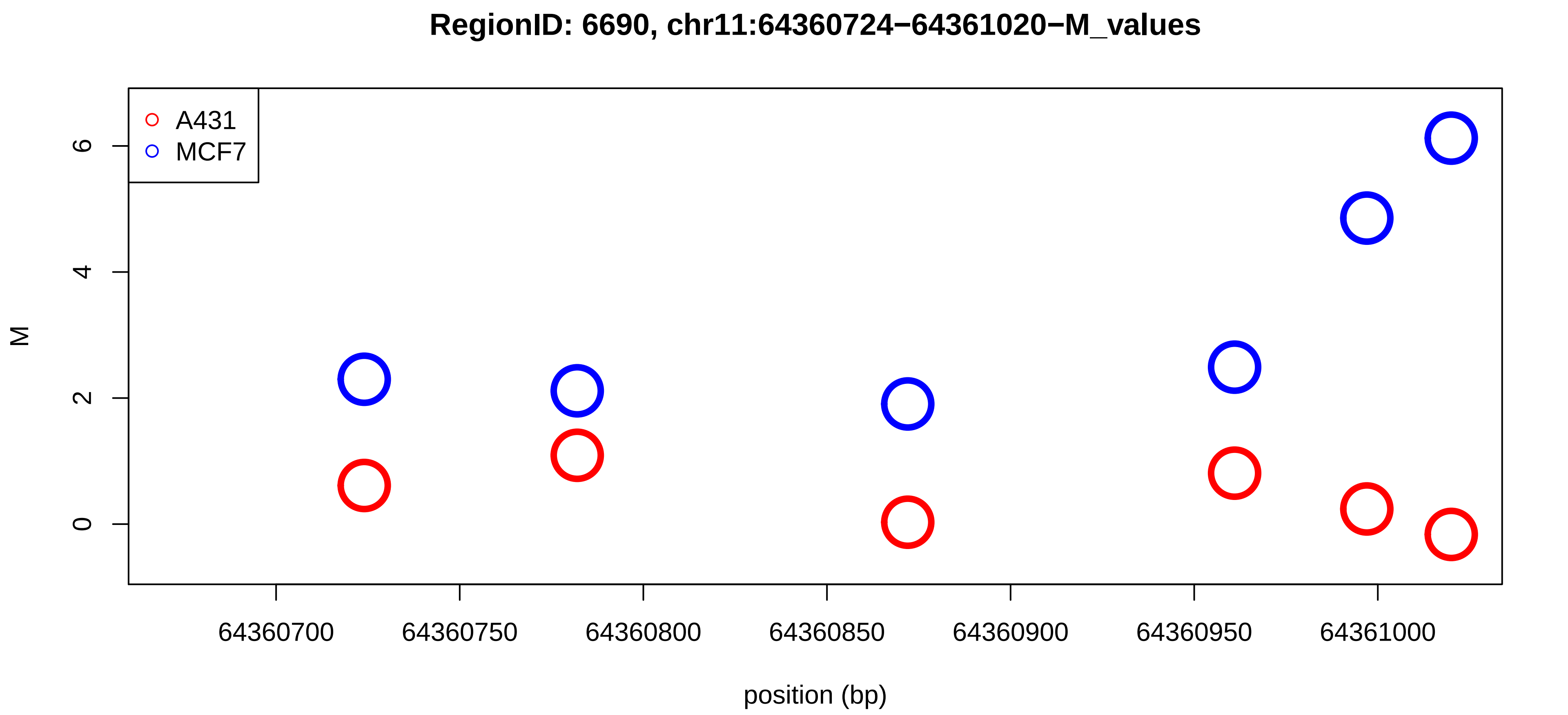
RegionID: 6690, chr11:64360724−64361020−M_values
A431
MCF7
M
0
2
4
6
64360700
64360750
64360800
64360850
64360900
64360950
64361000
position (bp)

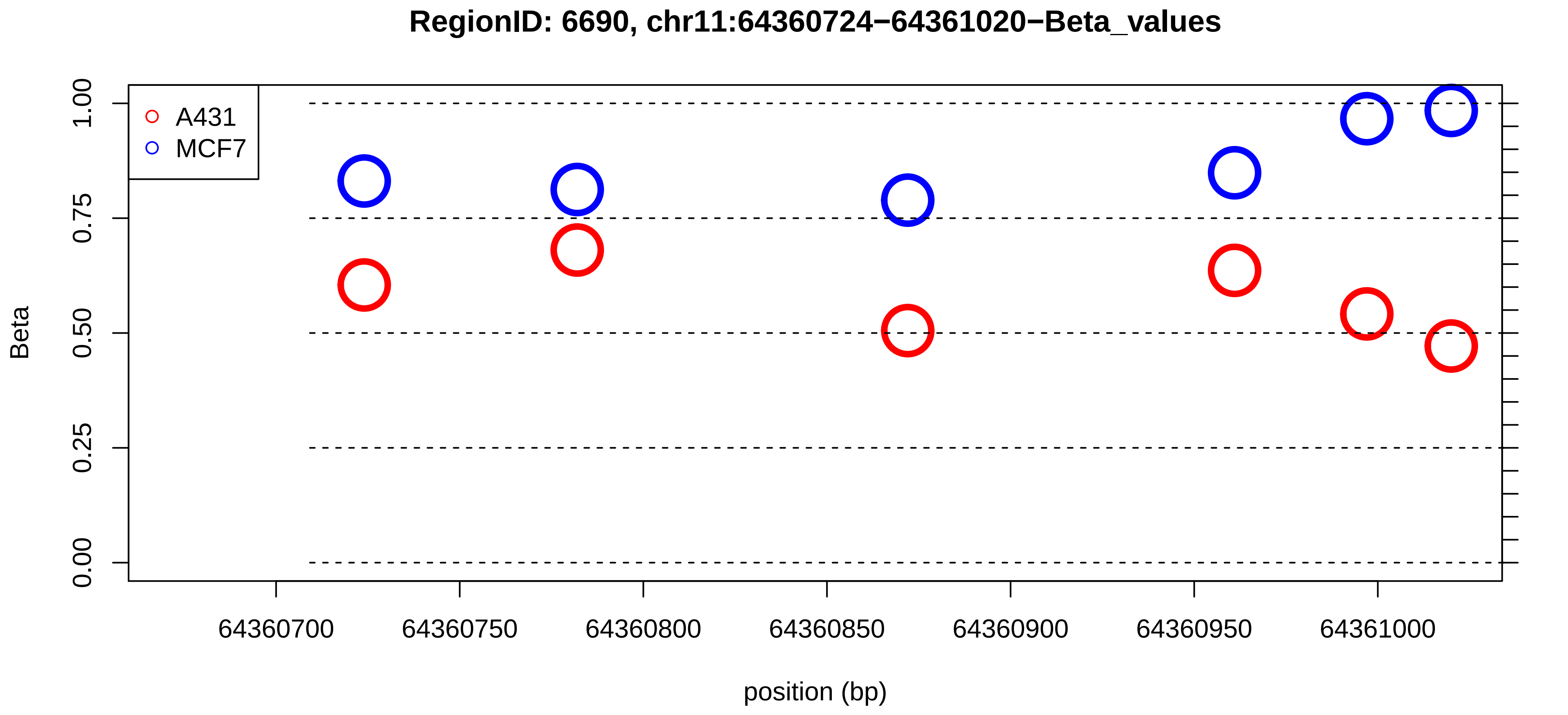
RegionID: 6690, chr11:64360724−64361020−Beta_values
A431
MCF7
Beta
0.00
0.25
0.50
0.75
1.00
64360700
64360750
64360800
64360850
64360900
64360950
64361000
position (bp)

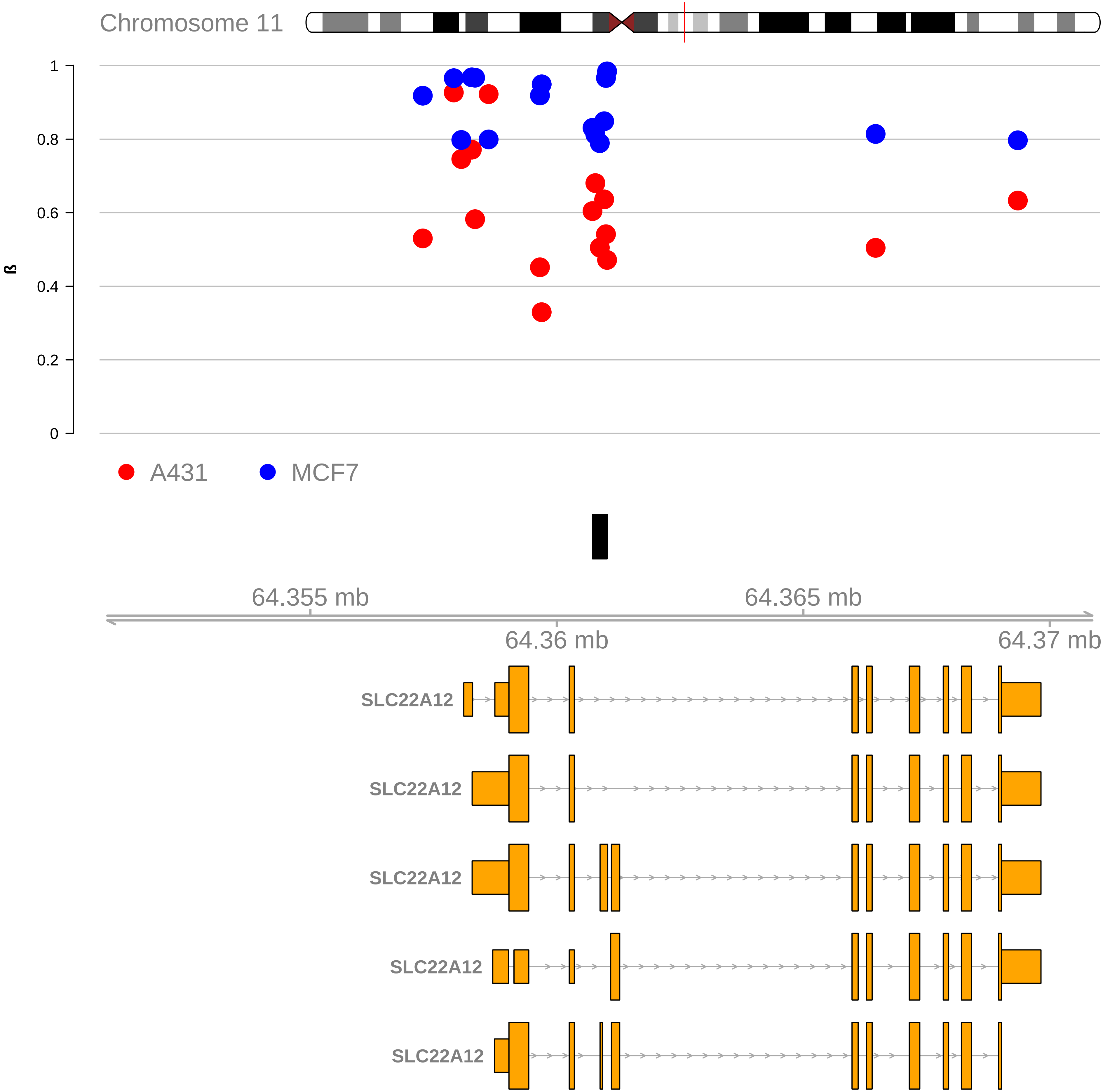
Chromosome 11
1
0.8
0.6
0.4
0.2
0
ß
A431
MCF7
64.355 mb
64.365 mb
64.36 mb
64.37 mb
SLC22A12
SLC22A12
SLC22A12
SLC22A12
SLC22A12
